# Scan patterns during scene viewing predict individual differences in clinical traits in a normative sample

**DOI:** 10.1371/journal.pone.0196654

**Published:** 2018-05-23

**Authors:** Taylor R. Hayes, John M. Henderson

**Affiliations:** 1 Center for Mind and Brain, University of California Davis, Davis, California, United States of America; 2 Department of Psychology, University of California Davis, Davis, California, United States of America; Bournemouth University, UNITED KINGDOM

## Abstract

The relationship between viewer individual differences and gaze control has been largely neglected in the scene perception literature. Recently we have shown a robust association between individual differences in viewer cognitive capacity and scan patterns during scene viewing. These findings suggest other viewer individual differences may also be associated with scene gaze control. Here we expand our findings to quantify the relationship between individual differences in clinical traits and scene viewing behavior in a normative sample. The present study used Successor Representation Scanpath Analysis (SRSA) to quantify the strength of the association between individual differences in scan patterns during real-world scene viewing and individual differences in viewer attention-deficit disorder, autism spectrum disorder, and dyslexia scores. The SRSA results revealed individual differences in vertical scan patterns that explained more than half of the variance in attention-deficit scores, a third of the variance in autism quotient scores, and about a quarter of the variance in dyslexia scores. These results suggest that individual differences in attention-deficit disorder, autism spectrum disorder, and dyslexia scores are most strongly associated with vertical scanning behaviors when viewing real-world scenes. More importantly, our results suggest scene scan patterns have promise as potential diagnostic tools and provide insight into the types of vertical scan patterns that are most diagnostic.

## Introduction

How we direct our attention in complex visual scenes has been studied since the pioneering eye tracking work of Buswell [1] and Yarbus [2]. Based on their findings, Buswell and Yarbus suggested that gaze control in complex visual scenes is a function of the current task goals, the properties of the scene stimulus, and the properties of the viewer [1, 2]. However, the vast majority of the scene literature has focused on the role of image properties and/or task goals [3], with little work on how viewer individual differences are related to scene gaze control [4, 5]. As a result, very little is known about the relationship between viewer individual differences and gaze control during active scene viewing.

**Fig 1:**
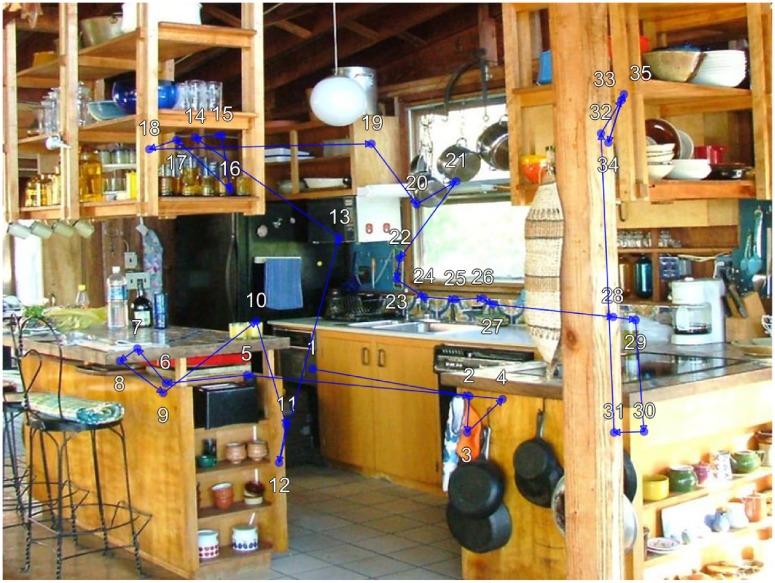
Example viewer scan pattern during scene memorization. The viewer was instructed to memorize the scene for a later memory test. During 12 seconds of viewing the viewer made 35 fixations. The blue circles show each fixation location and the blue lines indicate saccades between fixations. The white numbers indicate the sequential order of fixations 1 through 35.

We [4] recently used an individual differences approach to establish an association between individual differences in viewer cognitive capacities and scan patterns during active scene viewing. Scan patterns, also referred to as scanpaths, [6–8], refer to the sequential pattern of stimulus fixations (See Fig 1). Our findings revealed individual differences in scene scan patterns that explained more than 40% of the variance in viewer intelligence and working memory capacity, and more than a third of the variance in speed of processing [4]. Our findings also revealed that only sequential eye movement patterns were capable of explaining significant variance in cognitive capacity, while other common fixation measures such as fixation duration, saccade amplitude, and fixation density could not. Here we seek to expand this work to individual differences in clinical traits.

Attention-deficit disorder (ADD), dyslexia, and autism spectrum disorder (ASD) are pervasive conditions that have each been linked to atypical attentional control (for review see [9]). For example, studies using simplified visual stimuli such as attentional blink, Go/No go, and anti-saccade tasks have shown that ADD groups commit more errors [10–13] and are more vulnerable to distracting visual information [11, 13] than control groups. While dyslexia is a language-based learning disorder, recent findings suggest at least some cases may be associated with general attentional deficits rather than phonological processing deficits [14–16]. Finally, people with autism spectrum disorder have been shown to exhibit decreased attention to social stimuli and increased attention to non-social objects [17–19], and deficits in pursuit eye movements [20].

This previous body of work predominantly uses simple, controlled experimental stimuli to study atypical attentional control in ADD, ASD, and dyslexia. However, recent work [5] took a novel approach by examining atypical attentional control in people with ASD using complex, real-world scenes as stimuli. Specifically, they compared an ASD group to a control group, examining how their gaze allocation was explained by center/background bias, pixel-level saliency, object-saliency, and semantic-level saliency. The results showed that people with ASD have stronger image center bias regardless of scene content, increased pixel-level saliency, and decreased object- and semantic-level saliency relative to controls. More broadly, their findings suggest that complex, real-world scene stimuli may be better suited for understanding clinical atypical attention control than simple experimental stimuli and could potentially be used diagnostically as a screening tool [21].

In the present study, we examined the association between individual differences in clinical traits and individual differences in real-world scene scan patterns in a normative sample. We had two goals in the study. The first goal was to quantify the strength of the association between individual differences in clinical traits and scan patterns during real-world scene viewing in a normative sample. Achieving this goal will serve as a proof of concept that scan patterns can be used to identify individuals with higher/lower clinical trait scores. Second, we sought to define and visualize the scene scan patterns associated with higher and lower clinical trait scores. In particular, creating a representation of the scan patterns that are most diagnostic of high clinical scores might provide potential diagnostic markers for future clinical studies.

Following our recent work [4], we used *Successor Representation Scanpath Analysis* (SRSA, [22, 23]) to quantify the association between individual differences in clinical trait measures and scene scan patterns. SRSA uses temporal difference learning [24] to capture statistical regularities in scan patterns in a fixed-size matrix called a *successor representation* (SR, [25]) that can be aggregated across trials and analyzed with standard multivariate methods. SRSA was used to quantify the strength of the association between individual differences in scan patterns and individual differences in clinical traits by identifying individual differences in scan patterns during scene encoding that predicted viewers’ attention-deficit disorder, autism spectrum quotient, and dyslexia scores that were assessed via a separate clinical test battery.

Our results revealed that individual differences in clinical traits are associated with individual differences in scene scanning behavior in a normative sample. Specifically, SRSA identified individual differences in scan patterns during scene viewing that explained more than half of the variance in attention-deficit scores, a third of the variance in autism quotient scores, and just under a quarter of the variance in dyslexia scores. An analysis of the state spaces and scan pattern representations that were most predictive suggested that individual differences in clinical traits were most strongly associated with how viewers guided their attention vertically across the scenes.

## Materials and methods

### Participants

Seventy-nine University of South Carolina undergraduate students with normal or corrected-to-normal vision participated in the study. Data in the full study was collected from June 2013 to November 2013 and no participants dropped out (0/79). Our previous work [4] reported participants that completed cognitive capacity measures; here we report the participants that completed clinical trait measures for the first time (N = 40). The mean participant age was 20.2 (range 18–30). The study was approved by the University of South Carolina Institutional Review Board. All participants were naive concerning the purposes of the experiment and provided informed written consent.

### Apparatus

Eye movements were recorded with a SR Research EyeLink 1000 plus tower mount eye tracker (spatial resolution 0.01°) sampling at 1000 Hz [26]. Participants sat 80 cm away from a 21” monitor, so that scenes subtended approximately 29° x 22° of visual angle. Head movements were minimized using a chin and forehead rest. Although viewing was binocular, eye movements were recorded from the right eye. The experiment was controlled with SR Research Experiment Builder software [27].

### Scene stimuli and task procedure

Stimuli consisted of 40 digitized photographs of real-world scenes. The real-world scene stimuli included a variety of indoor and outdoor environments. Participants were instructed to memorize each scene in preparation for a later memory test that was not administered. Each trial began with a fixation on a cross at the center of the display for 300 msec. Following fixation, each scene was presented for 12 seconds while eye movements were recorded. Scenes were presented in the same order across all participants. After completing the scene memorization task, participants completed a series of clinical individual difference measures including pre-diagnostic screens for attention-deficit disorder, autism spectrum disorder, and dyslexia. Eye movements were not recorded for the clinical individual difference measures.

### Clinical individual difference measures

Individual differences in attention-deficit score were measured using the Jasper/Goldberg adult ADD (ADDAT) screen [28, 29]. The ADDAT is a screening instrument for adult ADD which consists of 24 questions that are answered using a 5-point Likert scale. ADDAT scores over 70 are associated with an increased likelihood of ADD. Individual differences in autism spectrum disorder (ASD) score were measured using the autism-spectrum quotient (AQ) test [30, 31]. The AQ test is a widely used screening instrument for ASD consisting of 50 questions that are answered using a 4-point Likert scale. AQ scores over 31 are associated with an increased likelihood of ASD. Individual differences in dyslexia score were accessed using the Vinegard revised adult dyslexia checklist [32, 33]. The dyslexia checklist consists of 20 yes/no questions. Dyslexia scores of 9 or more are associated with increased likelihood of dyslexia. The reported scores for each clinical individual difference measure is the sum across all items, where higher scores are indicative of increased likelihood of the disorder. However, it should be noted that these questionnaires are self-report screening tools and are not diagnostic on their own. A clinical diagnosis requires a detailed medical history and a complete battery of diagnostic tests.

### State space definitions

**Fig 2:**
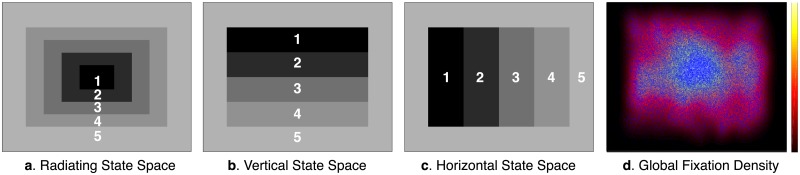
State spaces used to define sequential scan patterns during scene viewing. Scan patterns during scene viewing were defined by mapping fixation positions to 3 different state spaces. The Radiating state space (a) measured viewer tendencies to shift their overt attention between central and peripheral scene information. The Vertical and Horizontal state spaces (b and c) measured observers tendencies to shift their overt attention vertically and horizontally. Each of the state spaces contained an outside state 5 that reflected the center bias observed in the global fixation density (d) across all scenes and participants. Each state space was applied globally across all 40 scenes.

Following Hayes and Henderson (2017), three different state spaces were defined a priori to capture simple scene viewing tendencies and applied to eye movements to produce scan pattern sequences across each scene (See Fig 2). Each state space spanned the full display (1024 x 768 pixels) and was used to examine different patterns in how participants shifted their overt attention during scene viewing. The *Radiating* state space consisted of a series of radiating rectangular areas of interest (AOIs) from the scene center to the periphery, and was used to represent observers’ tendencies to shift their overt attention between more central and peripheral scene information. The *Vertical* state space consisted of 4 equal rectangular horizontal AOIs, and was used to represent observers’ tendencies to shift their overt attention vertically across each scene. The *Horizontal* state space consisted of 4 equal rectangular vertical AOIs, and was used to represent observers’ tendencies to shift their overt attention horizontally across each scene. Note each of these 3 state spaces contained an outside border that reflected the central fixation bias, the commonly observed phenomenon in which participants concentrate their fixations more centrally and rarely fixate the outside border of a scene [34]. A state number of 5 was chosen for each state space instead of 10, as was used in previous SRSA applications [22, 23], because there were too few fixations per scene viewing trial to support a state resolution of 10. The Radiating, Vertical, and Horizontal state spaces were each applied separately to all 40 scenes to map each participant’s fixation positions (i.e., X and Y coordinates) to 1 of the 5 distinct states within each state space.

### Eye movement data

A 13-point calibration procedure was performed at the start of each session to map eye position to screen coordinates. Successful calibration required an average error of less than 0.49° and a maximum error of less than 0.99°. Fixations and saccades were segmented with EyeLink’s standard algorithm using velocity and acceleration thresholds (30°/s and 9500°/s; [26]). The eye movement data was imported into Matlab using the EDFConverter tool, which converted the EyeLink data file to text that was then imported into Matlab. In Matlab, the eye movement data from each participant was inspected for excessive artifacts caused by blinks or loss of calibration due to incidental movement by examining the mean percent of signal across all trials [35, 36]. Fourteen participants with less than 75% signal were removed, leaving 65 participants that were tracked very well (mean signal = 91.74%). Traditional eye movement metrics such as fixation duration, saccade amplitude, and fixation number were computed for each trial. In addition, scan patterns across the 3 different state spaces (Fig 2) were computed using the X and Y gaze positions of each fixation within each scene trial. The first fixation of each trial was removed because it was always at the center of the display as a result of the pretrial fixation period, and thus uninformative.

### Successor Representation Scanpath Analysis

Successor Representation Scanpath Analysis (SRSA, [4, 22, 23]) was used to capture statistical regularities in eye movement scan patterns within each of the 3 different state spaces and predict individual differences in the clinical scores of the participants. SRSA quantifies regularities in scan patterns using temporal-difference learning [24] to construct a fixed-size matrix called a *successor representation* (SR, [25]). The key idea behind SRSA is that upon observing a scan transition from one state to another, instead of simply updating the transition probability from the first to the second state, SRSA associates the first state with the second state and all expected subsequent states based on prior visits to the second state. A successor representation [25] was calculated for each trial scan pattern, resulting in one 5 × 5 SR matrix ***M*** per trial for each participant. To calculate the trial SR matrix, each trial SR matrix is initialized with zeros and then updated for each transition in the scan pattern sequence. Consider a transition from state *i* to state *j*. The *i*th column of the matrix—the column corresponding to the “sender” state—is updated according to Eq (1):

$$\begin{array}{c} \Delta M_{i}=\alpha \left(I_{j}+\gamma M_{j}-M_{i}\right), \end{array}$$ (1)

where ***I*** is the identity matrix, each subscript picks a column in a matrix, *α* is a learning-rate parameter (0 < *α* < 1), and *γ* is a temporal discount factor (0 < *γ* < 1). The learning rate parameter *α* controls the incremental updating and *γ* controls the amount of temporal discounting. The *γ* parameter is the key to extending the temporal step size to encompass both immediate and long-range transitions—it includes the discounted future states in the prediction from the current state. After traversing a scan pattern for a given scene, the resulting successor representation can be conceptualized as having extracted the statistical regularities in temporally extended scan patterns. Specifically, an SR matrix contains, for each state, the temporally discounted number of expected future fixations to all states [25]. Given the uniform size of SRs and a commonly defined set of states, the SR matrices from different observers and/or trials can be analyzed using standard statistical methods to identify significant pattern regularities for various comparisons of interest. SRSA has previously been successfully applied to study individual differences in problem solving strategies during matrix reasoning [22, 37], the role of strategy refinement in pre-post designs using matrix reasoning tests [23], and the relationship between individual differences in cognitive capacity and scan patterns during scene perception [4].

In the present study, SRSA was used to quantify the relationship between individual differences in clinical trait measures (attention-deficit disorder, autism spectrum disorder, and dyslexia scores) and scan patterns during real-world scene viewing. Briefly summarized, for each state space definition shown in Fig 2, a successor representation (SR) was calculated for each scene scan pattern, the mean across the 40 scene SRs for each participant was computed, and then individual differences in the mean participant SRs (reduced by principal component analysis to 5 dimensions) were used to predict each clinical measure using multiple regression. For all SRSA analyses a goodness-of-fit *R*^2^ across all participants and a leave-one-out cross-validated $R_{cv}^{2}$ fit are reported. The cross-validated fit is a much better estimate of the generalization performance than the goodness-of-fit *R*^2^ [38–40]. The goodness-of-fit *R*^2^ is inflated because it reflects not only genuine regularities in the population, which will generalize to new cases, but also the idiosyncrasies of the training sample, which will not. SRSA was systematically performed in this same way for each state space definition (i.e., Radiating, Vertical, and Horizontal) to predict participant clinical individual difference scores.

### Procedure for interpreting SRSA weights

One of the major advantages of SRSA is that the prediction weight matrices and principal components are interpretable [22]. The main barrier to this interpretation is providing a way to distill and visualize the higher-order sequential patterns that are being captured —a notoriously difficult visualization task [41]. In order to assist in the interpretation of the higher-order sequential patterns captured by the SRSA prediction weight matrices in the less constrained scene encoding task, a general procedure was developed to identify the most illustrative scene scan patterns for each SRSA individual difference model (i.e., the individual scene scan patterns that were the most highly positively and negatively correlated with the SRSA prediction weights).

A simple procedure was used to search for the most illustrative scan patterns for each clinical trait SRSA model. For each clinical trait measure, the participants with the 10 highest and 10 lowest scores were selected and the optimal SRSA parameters (*α* and *γ*) were used to convert their trial scan patterns into trial SRs. The correlation between the trial SRs from the 10 highest/lowest scoring participants were then correlated with the mean cross-validation prediction weight matrix from their respective SRSA model. These correlations represented on a trial-by-trial basis the association strength between the SRSA prediction weights and the trial scan patterns, where positive correlations were indicative of higher clinical trait scores and negative correlations were indicative of lower clinical trait scores. The trial with the greatest difference between the highest/lowest participants trial SR correlations was selected to help visualize high and low scoring scan patterns for each clinical trait measure within a common scene trial.

## Results

**Fig 3:**
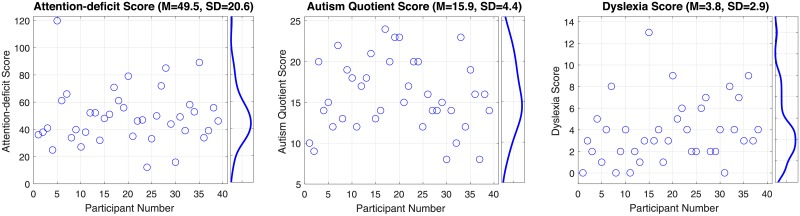
Observed clinical score scatter plots with corresponding probability density histograms. Clinical scores are shown for each clinical measure (ADD, autism, and dyslexia), where each point represents an individual participant score.

The 40 participants that completed the scene encoding task, met the eye tracking signal criterion, and completed clinical trait measures produced a total of 58,228 fixations with an average of 1,456 (SD = 176) fixations per participant. The mean participant fixation duration across all scene trials was 281 msec (SD = 41.2 msec). The mean participant saccade amplitude was 3.6° (SD = 0.38°) and the mean participant fixation number per scene trial was 37.4 (SD = 4.4). Fig 3 shows the mean, standard deviation, and the distribution of participant scores on the attention-deficit, autism quotient, and dyslexia measures. While clinical questionnaires help discriminate between clinical groups and typical controls, there is considerable variability in non-clinical populations as is evident in Fig 3. This variability in normative populations reflects how individuals vary along a continuum of the clinical traits measured by each screening instrument.

### Scene scan patterns and individual differences in clinical traits

Recall that our first goal was to quantify the strength of the association between individual differences in clinical trait measures and scene scan patterns. The goodnesss-of-fit and cross-validated SRSA prediction performance is shown in Table 1 for each clinical individual difference measure and state space combination. The SRSA results showed that individual differences in scan patterns could explain large amounts of variance in clinical scores. The Vertical state space produced the best prediction across all three clinical trait measures (ADD = $R_{cv}^{2}=0.53$; ASD = $R_{cv}^{2}=0.33$; dyslexia = $R_{cv}^{2}=0.23$). This suggests that vertical scene scanning behavior is more diagnostic of our clinical individual difference measures than horizontal or center to periphery scanning behavior. More broadly, the SRSA prediction results establish and quantify the association between individual differences in scan patterns during scene viewing and the underlying individual differences in clinical traits in ADD, ASD, and dyslexia.

**Table 1:** Successor Representation Scanpath Analysis (SRSA) results: Goodness-of-fit *R*^2^ and leave-one-out cross-validation ($R_{cv}^{2}$) for predicting individual differences (ID) from scan pattern regularities for all 3 state spaces (Radiating, Vertical, and Horizontal). An asterisk highlights the most predictive SRSA models for each clinical measure that are discussed in detail in the results section and shown in Figs 4, 5, and 6.

	Radiating State Space				Vertical State Space				Horizontal State Space			
ID measures	R^2^	R^2^_cv_	*γ*	*α*	R^2^	R^2^_cv_	*γ*	*α*	R^2^	R^2^_cv_	*γ*	*α*
Adult Attention-deficit Score	0.57	0.23	1.00	0.22	0.80	0.53*	0.67	0.43	0.68	0.23	0.96	1.00
Autism Quotient Score	0.57	0.26	0.98	0.03	0.62	0.33*	0.21	1.00	0.46	0.05	0.89	0.86
Dyslexia Score	0.61	0.13	0.99	1.00	0.62	0.23*	0.95	0.88	0.52	0.14	0.01	0.21

**Fig 4:**
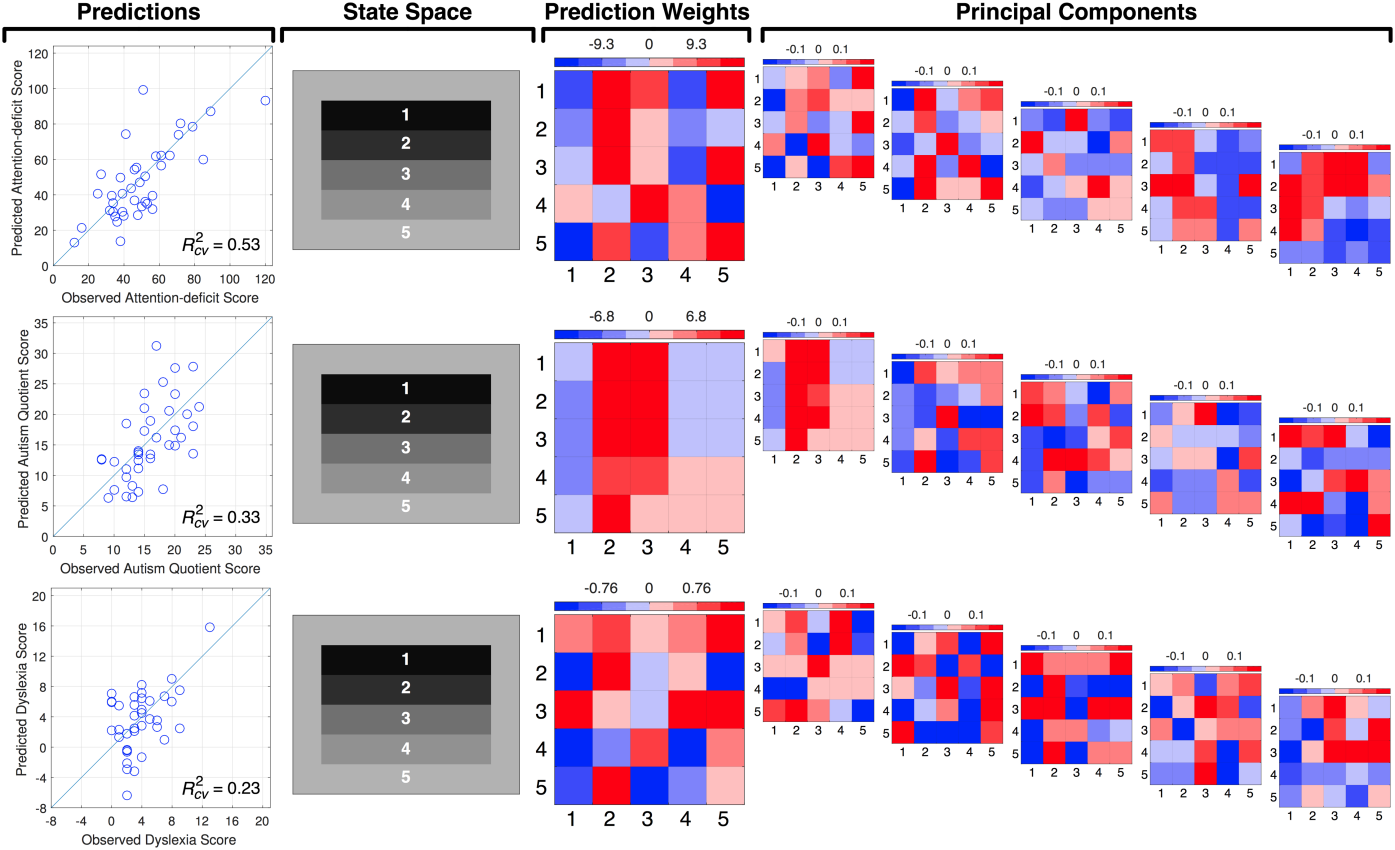
Clinical score observations and predictions, state space, prediction weights, and principal components for each SRSA cross-validated model. The ‘Predictions’ column shows the observed and SRSA predicted clinical scores and their squared correlation, where the line represents a squared correlation of 1. The state space column shows the state space definition for each model. The ‘Prediction Weights’ column shows the mean prediction weights across the leave-one-out fits for each individual difference measure. Finally, the 5 mean principal components across the leave-one-out fits are shown for each cross-validated SRSA model ranked according to the mean amount of variance they captured across the training sets. Positive values associated with higher individual difference scores are shown in red and negative values associated with lower individual difference scores are shown in blue. In the prediction weights and principal component matrices the x-axis represents the sender state and the y-axis represents the receiver state.

**Fig 5:**
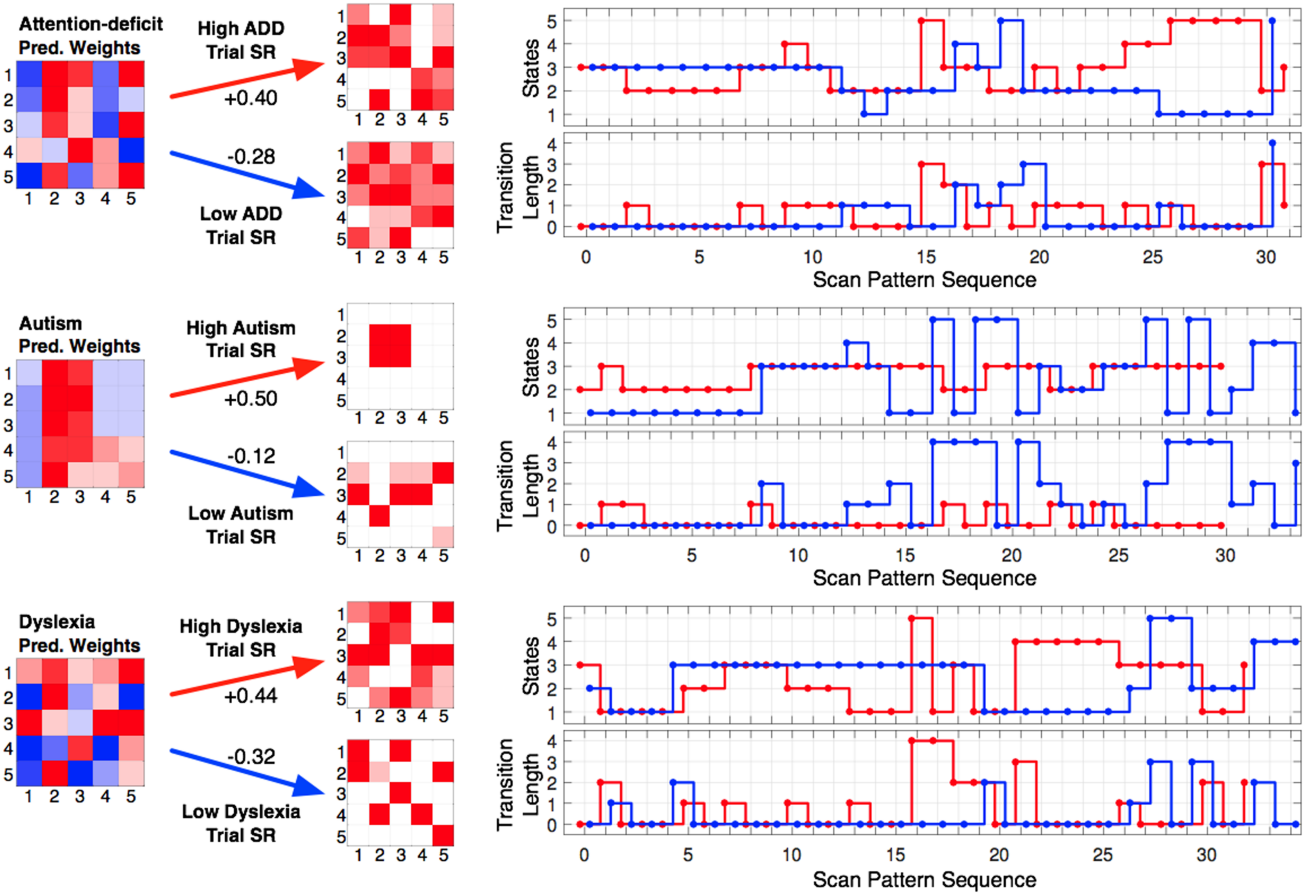
Illustrative scan patterns for for each clinical trait SRSA model in state space. The mean cross-validated prediction weights for the best SRSA model are shown for each clinical measure. Illustrative scene state transitions that were most strongly positively (red) and negatively (blue) correlated with the prediction weights and their corresponding trial SRs are shown to the right of each set of prediction weights. The top scan pattern panel shows the state transitions at each sequential position and the bottom scan pattern figure shows the transition length of each state transition. In the prediction weights and trial SR matrices the x-axis represents the sender state and the y-axis represents the receiver state.

**Fig 6:**
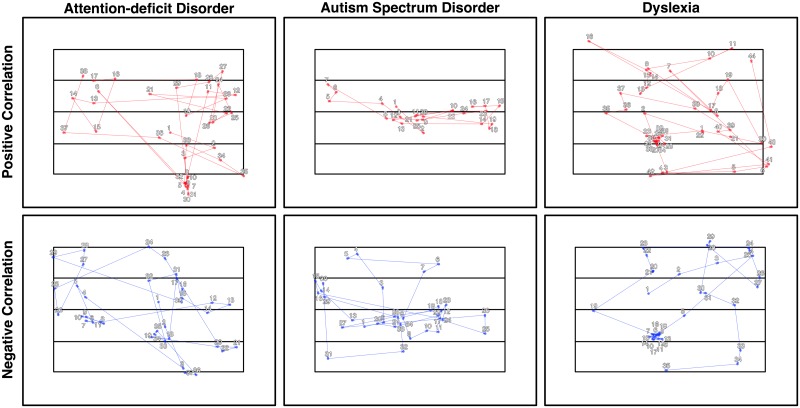
Illustrative scan patterns for each clinical trait SRSA model shown as fixations in image space. The illustrative trial scan patterns that were most strongly positively (top row, red) and negatively (bottom row, blue) correlated with the prediction weights from Fig 5 are shown as their corresponding fixations within the common scene space for each clinical trait. The numbers indicate the sequential order of fixations within each scan pattern.

With an association established, our second goal was to try to gain insight into the scan pattern differences during scene viewing that distinguish between individuals’ clinical trait scores. Fortunately, the SRSA prediction weights and principal components can be used for this purpose [4, 22, 23]. Fig 4 shows the mean prediction weights and mean principal components for the cross-validated SRSA model with the best prediction for each clinical trait measure highlighted with an asterisk in Table 1. Recall the mean cross-validated prediction weights are the sum of the principal components scaled by their respective regression coefficients and provide a summary of the 5 principal components. For the sake of simplicity and brevity, we will focus our interpretation on the SRSA model prediction weights. To assist in visualizing the patterns captured by the prediction weights, the most positively and negatively correlated trial scan patterns are also shown in Fig 5 as a state transition plot and in Fig 6 as the fixations in image space.

### Attention-deficit disorder

Attention-deficit scores were the best predicted by scene encoding scan patterns. Vertical scene scan patterns accounted for more than half the variance in ADD scores ($R_{cv}^{2}=0.53$). The best fitting gamma parameter was *γ* = 0.67. Recall that as *γ* moves toward 1, the relevant scan patterns become more temporally extended across multiple state transitions. The ADD prediction weight matrix in Fig 4 indicated higher ADD score was characterized by a bias toward scanning states 2 and 3, and shorter state transitions. These scan pattern regularities can also be seen in the ideal high and low ADD score example scan patterns shown in Figs 5 and 6.

### Autism spectrum disorder

Autism quotient scores were also well predicted, with the vertical cross-validated SRSA model accounting for a third of the variance ($R_{cv}^{2}=0.33$) with a *γ* of 0.21. ASD had the most readily interpretable prediction weights of the 3 clinical trait measures. Higher ASD scores were predicted by a strong scan pattern bias to move between and/or remain within states 2 and 3. In Fig 4 this is indicated by the strong red bands across state 2 and 3, and is also clearly evident in the illustrative high ASD scan pattern example in Figs 5 and 6.

### Dyslexia

Dyslexia had the weakest prediction of our clinical trait measures ($R_{cv}^{2}=0.23$) and was also the hardest to interpret. The best fitting *γ* value was nearly 1 (*γ* = 0.95) suggesting the relevant scan patterns were occurring across nearly the entire scene scan pattern. A comparison of the dyslexia and attention-deficit prediction weights shows a similar pattern. The correlation between them (*R* = 0.41) confirmed the visual overlap.

## Discussion

The effects of stimulus properties and task goals on scene viewing have dominated the scene perception literature. As a result, the relationship between viewer properties and scene gaze control remains poorly understood. Here we expanded upon our recent work [4] and used Successor Representation Scanpath Analysis (SRSA, [22]) to extract individual differences in scene scan patterns that predicted individual differences in viewers’ ADD, autism, and dyslexia scores in a normative sample. This method allowed us to quantify and build a representation of the association between individual clinical scores and scene viewing behavior. The results showed that individual differences in vertical scene scanning behaviors are most associated with individual differences in ADD ($R_{cv}^{2}=0.53$), autism ($R_{cv}^{2}=0.33$), and dyslexia ($R_{cv}^{2}=0.23$) scores. These exploratory findings make a number of contributions.

First, our results provide additional evidence that a complete model of scene gaze control should take viewer properties into account. While there have been an increasing number of scene gaze models suggesting important roles for saliency, [42, 43], semantic meaning [44], and information sampling [45], we are not aware of any model of scene gaze control which explicitly models viewer individual differences. In conjunction with our previous work [4], we have shown that individual differences in intelligence, working memory capacity, speed of processing, and now clinical traits are associated with individual differences in broad viewing behaviors during scene encoding. Our individual difference findings are mirrored by a recent study in which a large sample of fraternal and identical twins indicated increased spatial and temporal similarity in scene viewing behavior in identical twins [46]. Theoretically, these findings suggest that Buswell [1] and Yarbus [2] were correct, and a complete theory of scene gaze control should be a function not just of the stimulus and task, but also the properties of the viewer.

Second, our results offered insight into the association strength between scene viewing and each clinical measure, and a tangible representation of the scene scan patterns that are associated with higher and lower ADD, autism, and dyslexia scores. Broadly, SRSA was able to more accurately predict clinical trait measures that are more directly associated with atypical attention (e.g., ADD [12, 47, 48] and autism [49, 50]) than those which are thought to be more heterogeneous (e.g., dyslexia; [51, 52]). That is, we hypothesize that dyslexia scores had the weakest association with scene scan patterns due to the heterogeneity of the disorder and because it is a language-based disorder [53, 54]. The prediction performance we did observe in the dyslexia weights appeared similar to the ADD weights (although noiser), and may be driven by the comorbidity between dyslexia and ADD [55, 56]. To varying degrees, higher clinical scores were associated with vertical scene scan patterns that were biased toward the center third of the scene (states 2 and 3). The fact that this bias was stronger in the autism SRSA weights may be related to difficulties disengaging attention previously observed in other paradigms [57, 58] and scene viewing [5]. Future targeted work will be needed to test these hypotheses.

In agreement with [4], our results also revealed that scan patterns were more informative than the location, frequency, and/or duration of eye movements for understanding the role of viewer individual differences during scene viewing. The traditional eye metric model (See S3 Appendix and S2 Table) that used the mean and standard deviation in fixation duration, frequency, and saccade amplitude, could not account for significant variance in clinical scores. It was only when considering the extended sequential patterns between scene fixations that we were able to extract information on how gaze control was correlated with the underlying individual differences in the cognitive capacities of the viewers. Therefore, our results suggest not only that viewer properties are an important component of gaze control during scene encoding, but that they also reinforce spatio-temporal scan pattern dynamics as a critical target measure to test future models of scene gaze control.

Finally, it is worth comparing the state space performance for predicting individual differences in cognitive capacity scores [4] versus clinical scores. The vertical state space which was the best for predicting all 3 clinical trait measures was also the best for predicting a number of cognitive capacity scores including speed of processing (trail A and trail B scores), crystallized intelligence (SAT score), and working memory capacity (reading span score). This raises the question of whether cognitive capacity mediates the relationship between clinical scores and scan patterns. To determine the strength of the association between the clinical trait measures and the cognitive capacity measures [4], we computed the squared correlation between each measure and found no evidence of a relationship (See S2 Appendix and S1 Table). But the success of the vertical state space does suggest vertical scan patterns may be particularly relevant for understanding individual differences in scene viewing behavior. The one cognitive capacity that was poorly explained by the vertical state space in [4] was fluid intelligence (Raven Advanced Progressive Matrices scores), for which the radiating state space was by far the best. In our clinical trait measures, the radiating and horizontal state space performed similarly for ADD and dyslexia measures, but the radiating state space did show an advantage for predicting autism quotient score relative to the horizontal state space.

While our study represents an initial step toward understanding the relationship between individual differences in scan patterns and individual differences in clinical traits during scene perception, it is also limited in a number of ways. First, while our data show an association between individual differences in scan patterns and individual differences in clinical traits, it remains an open question what is driving this association [4]. A second limitation of our study is participants only completed a scene encoding task, and so it is unclear whether task demands modulate the strength of the relationship between individual differences in scan patterns and our clinical individual difference measures. It could be that another scene task (e.g., visual search or aesthetic judgment) or another task altogether (e.g., a reading task for dyslexia) may provide more discriminating scan patterns. Third, we examined individual differences in ADD, autism, and dyslexia scores among a general undergraduate population rather than a clinical population. The results may be even clearer and potentially diagnostic if the SRSA models were optimized to classify clinical patients relative to controls by replacing the multiple regression with a support vector machine classifier. Finally, we examined only 3 different potential state spaces (i.e., Radiating, Vertical, Horizontal) that defined and captured individual differences in broad scanning tendencies across all scenes. There may be more informative state space definitions that were not considered here. In future work it would be useful to explore other conceptualizations of scanning behavior such as scene-specific state spaces that use either visual saliency [43], scene meaning [44], or scene semantics [5, 59] to define the states within each scene. This could provide important information about how bottom-up image-based visual saliency and/or scene semantics interact with individual differences in scan patterns and viewer properties.

In summary, we found that individual differences in scan patterns during scene encoding predicted individual differences in viewer attention-deficit, autism quotient, and dyslexia scores in a normative sample. Broadly, these findings suggest an important new link between individual differences in gaze control during scene viewing and individual differences in clinical traits even in normative populations. An important direction for future work will be trying to determine the precise nature of this association, and integrating how viewer individual differences interact with bottom-up image-based properties, scene knowledge, and top-down task demands.

## Supporting information

S1 Appendix Determination of sample size. (PDF)

S2 Appendix Squared correlation between clinical and cognitive measures. (PDF)

S3 Appendix Traditional and transition probability models. (PDF)

S1 Table Squared correlation between clinical and cognitive measures. Clinical trait and cognitive capacity squared correlation matrix. The matrix shows the squared correlation (*R*^2^) between each clinical and cognitive measure followed by the number of subjects in parentheses. The abbreviated measures *Ospan* and *Rspan* indicate operation span and reading span respectively.
(PDF)

S2 Table Traditional model results. Goodness-of-fit and leave-one-out cross-validated performance for predicting clinical individual difference measures using traditional eye metrics. The traditional eye metric model included the mean and standard deviation of fixation duration, saccade amplitude, and fixation number as predictors in a multiple regression model to predict each participant’s clinical trait score. The results revealed that traditional eye metrics are poor predictors of individual differences in the clinical traits we measured.
(PDF)

S3 TableFirst-order transition model results.The Goodness-of-fit *R*^2^ and leave-one-out cross-validated ($R_{cv}^{2}$) for predicting individual differences (ID) in clinical trait measures from scan patterns using first-order transition frequency instead of successor representation. A comparison with the SRSA performance in Table 1 shows that successor representation provides an average increase in generalization performance ($R_{cv}^{2}$) of 324% (median 150%) relative to first-order transition model.(PDF)
