# Supplementary material for: Scan patterns during scene viewing predict individual differences in clinical traits in a normative sample
Source: PLoS One. 2018 May 23;13(5):e0196654. doi: 10.1371/journal.pone.0196654 (PMC5965850; doi:10.1371/journal.pone.0196654)
Supplement: S1 Appendix — (PDF) [file pone.0196654.s001.pdf]

S1 Appendix. Determination of sample size.

The target sample size necessary for SRSA was determined based on previous work using scan patterns and Successor Representation Scanpath Analysis (SRSA) to predict individual differences in cognitive capacities [4,22,23]. These studies indicated that a relatively small number of participants (N=30-40) can be used to train and validate a predictive SRSA model. Previous work [29,31,33] has also shown that the reliability of the clinical trait measures is good (Cronbach's alpha > 0.80) and supports a sample size in this range. Therefore, we aimed to collect eye movement data and clinical scores so that each clinical trait measure had approximately 40 participants.

Since all of our analyses were focused on quantifying how well scan patterns (and other eye metrics) can predict clinical scores, the main risk that must be accounted for in the predictive models based on these samples is overfitting. In order to control for overfitting and provide estimates of how well each predictive model will generalize to new data, leave-one-out cross-validation [38,39] was performed and is reported for all analyses. Finally, while the clinical trait measures have high reliability, this still limits the observable correlation between variables [40]. Therefore, our findings may underestimate the true correlation between clinical trait scores and scene scan patterns.
