# Supplementary material for: Scan patterns during scene viewing predict individual differences in clinical traits in a normative sample
Source: PLoS One. 2018 May 23;13(5):e0196654. doi: 10.1371/journal.pone.0196654 (PMC5965850; doi:10.1371/journal.pone.0196654)
Supplement: S2 Appendix — (PDF) [file pone.0196654.s002.pdf]

S2 Appendix. Squared correlation between clinical and cognitive measures.

The squared correlation between the clinical trait measures and the cognitive capacity measures previously reported [4] is shown in S1 Table. The results show that the clinical trait measures we used share a relatively small amount of variance with cognitive capacity measures (M=0.03, SD=0.04). This suggests that the clinical SRSA models are explaining variance specific to the clinical traits we assessed and not the underlying cognitive capacity of the participants. It also accounts for why the clinical SRSA prediction weights are different than the cognitive SRSA prediction weights [4] even when the best state spaces are the same (e.g., the vertical state space was best for a number of cognitive capacity measures including trail A, trail B, SAT score, and reading span score).
