# Supplementary material for: Scan patterns during scene viewing predict individual differences in clinical traits in a normative sample
Source: PLoS One. 2018 May 23;13(5):e0196654. doi: 10.1371/journal.pone.0196654 (PMC5965850; doi:10.1371/journal.pone.0196654)
Supplement: S3 Appendix — (PDF) [file pone.0196654.s003.pdf]

S1 Table. Squared correlation between clinical and cognitive measures.

	**ADD**	**ASD**	**dyslexia**	**Raven's**	**SAT**	**Trail A**	**Trail B**	**Ospan**	**Rspan**
ADD	—								
ASD	0.05 (38)	—							
dyslexia	0.06 (38)	0.10 (37)	—						
Raven's	0.02 (39)	0.01 (39)	0.17 (38)	—					
SAT	0.05 (20)	0.00 (20)	0.03 (19)	0.11 (20)	—				
trail A	0.01 (39)	0.00 (39)	0.06 (38)	0.02 (40)	0.00 (20)	—			
trail B	0.01 (39)	0.00 (39)	0.05 (38)	0.15 (40)	0.04 (20)	0.37 (40)	—		
Ospan	0.00 (37)	0.06 (37)	0.01 (36)	0.08 (38)	0.00 (19)	0.01 (38)	0.08 (38)	—	
Rspan	0.05 (38)	0.01 (38)	0.02 (37)	0.01 (39)	0.04 (19)	0.21 (39)	0.34 (39)	0.04 (37)	—
