# Supplementary material for: Scan patterns during scene viewing predict individual differences in clinical traits in a normative sample
Source: PLoS One. 2018 May 23;13(5):e0196654. doi: 10.1371/journal.pone.0196654 (PMC5965850; doi:10.1371/journal.pone.0196654)
Supplement: S1 Table — Clinical trait and cognitive capacity squared correlation matrix. The matrix shows the squared correlation (R2) between each clinical and cognitive measure followed by the number of subjects in parentheses. The abbreviated measures Ospan and Rspan indicate operation span and reading span respectively. (PDF) [file pone.0196654.s004.pdf]

S3 Appendix. Traditional and transition probability models.

SRSA performance was compared to two simpler eye movement data models: a traditional eye metric model and a first-order transition probability model. The traditional eye metric model calculated the mean and standard deviation for fixation duration, saccade amplitude, and fixation number for each participant across all 40 scenes. These 6 eye metrics were then used as predictors in a multiple regression model to predict clinical trait scores. To allow a direct comparison between the traditional eye metric model performance and the SRSA model performance, both a goodness-of-fit R^2 and leave-one-out cross-validation (R^2_{cv}) were computed for each clinical trait measure.

The transition probability model computed first-order transition probabilities for the same 3 state spaces that were used for each SRSA model (i.e., Radiating, Vertical, and Horizontal). The only difference between the transition probability models and the SRSA models is that a first-order transition matrix was computed for each trial scan pattern rather than a successor representation. The same temporal difference learning rate parameter α, dimensionality reduction using PCA, and cross-validation procedures were performed for the first-order transition probability models. The comparison between the SRSA models and the transition probability models provides a direct estimate of the gains in prediction performance that are due to the temporal difference learning rate and PCA dimensionality reduction, versus the performance gains that are due to the power of the successor representation to extract temporally extended scan pattern regularities beyond first-order transitions.

The traditional eye movement model results for each individual difference measure are shown in S2 Table. The traditional eye metric model using mean and standard deviation of fixation duration, saccade amplitude, and fixation number as predictors and was only able to account for a small amount of variance in dyslexia score. S3 Table shows the performance of the first-order transition model that used an identical prediction algorithm to the SRSA models. While the first-order transition model was able to successfully explain some of the variance in individual difference measures, a comparison with the SRSA model performance revealed that on average successor representation increased generalization performance by 324% (median=150%). These results highlight the importance of not collapsing across the temporal dynamics of eye movements and the benefit of extending the temporal boundary beyond just first-order transitions by using successor representation to capture temporally extended regularities in scan patterns. Finally, it is worth noting the importance of performing cross-validation to test models of eye movement data. Consistent with previous modeling of eye movement data [4,22,23,37], the SRSA, first-order, and traditional eye metric model results all show the goodness-of-fit R^2 is consistently inflated due to overfitting. Our results support the general recommendation that statistical models of eye movement data should be cross-validated to provide more accurate estimates of their ability to generalize to new data.
