# Supplementary material for: Scan patterns during scene viewing predict individual differences in clinical traits in a normative sample
Source: PLoS One. 2018 May 23;13(5):e0196654. doi: 10.1371/journal.pone.0196654 (PMC5965850; doi:10.1371/journal.pone.0196654)
Supplement: S2 Table — Goodness-of-fit and leave-one-out cross-validated performance for predicting clinical individual difference measures using traditional eye metrics. The traditional eye metric model included the mean and standard deviation of fixation duration, saccade amplitude, and fixation number as predictors in a multiple regression model to predict each participant’s clinical trait score. The results revealed that traditional eye metrics are poor predictors of individual differences in the clinical traits we measured. (PDF) [file pone.0196654.s005.pdf]

S2 Table. Traditional model results

**Individual difference measures**	$\mathbf{R^2}$	$\mathbf{R^2_{cv}}$
Adult Attention-deficit Score	0.16	0.01
Autism Quotient Score	0.03	0.00
Dyslexia Score	0.30	0.05
