# Supplementary material for: Scan patterns during scene viewing predict individual differences in clinical traits in a normative sample
Source: PLoS One. 2018 May 23;13(5):e0196654. doi: 10.1371/journal.pone.0196654 (PMC5965850; doi:10.1371/journal.pone.0196654)
Supplement: S3 Table — The Goodness-of-fit R2 and leave-one-out cross-validated (Rcv2) for predicting individual differences (ID) in clinical trait measures from scan patterns using first-order transition frequency instead of successor representation. A comparison with the SRSA performance in Table 1 shows that successor representation provides an average increase in generalization performance (Rcv2) of 324% (median 150%) relative to first-order transition model. (PDF) [file pone.0196654.s006.pdf]

S3 Table. First-order transition model results.

	**Radiating State Space**			**Vertical State Space**			**Horizontal State Space**		
**ID Measures**	$\mathbf{R^2}$	$\mathbf{{R^2}_{cv}}$	$\boldsymbol{\alpha}$	$\mathbf{R^2}$	$\mathbf{{R^2}_{cv}}$	$\boldsymbol{\alpha}$	$\mathbf{R^2}$	$\mathbf{{R^2}_{cv}}$	$\boldsymbol{\alpha}$
Adult Attention-deficit Score	0.50	0.04	0.01	0.56	0.08	0.20	0.45	0.06	0.03
Autism Quotient Score	0.54	0.14	0.57	0.59	0.24	0.05	0.44	0.02	0.07
Dyslexia Score	0.45	0.01	0.24	0.56	0.10	0.43	0.51	0.15	0.22
